# Clinical and Radiographic Evaluation of Third-Generation Pericardium Membrane for the Treatment of Grade II Furcation Defect in Stage III Periodontitis Patients

**DOI:** 10.3390/medicina59030572

**Published:** 2023-03-15

**Authors:** Mohamed O. Elboraey, Eihab A. Mously

**Affiliations:** 1 Oral Medicine, Periodontology, Oral Diagnosis and Radiology Department, Faculty of Dentistry, Tanta University, Tanta 31527, Egypt; 2 Department of Preventive Dental Sciences and Periodontology, College of Dentistry, Taibah University, Madinah 41411, Saudi Arabia

**Keywords:** 3rd generation, CBCT, ITK-SNAP, pericardium, two-layered

## Abstract

*Background and Objectives:* Guided tissue regeneration, with or without a bone graft, is a modality for the treatment of furcation involvement. Because the direct application of a bone graft into the periodontal defect has drawbacks, such as the risk of microbial contamination and/or graft containment, a new modality of directly loading bone graft particles over the barrier membrane is now used. This study aimed to evaluate clinically and radiographically the effects of a two-layered membrane consisting of a layer of nanohydroxyapatite particles on a pericardium membrane in the treatment of stage III periodontitis, compared with direct application of a nanohydroxyapatite bone graft. *Materials and Methods*: Forty individuals with grade II furcation involvement were divided into two groups. Group I was treated with a two-layered membrane consisting of a pericardium membrane with nanohydroxy particles loaded onto its surface; group II was treated with direct application of a nano bone graft covered with pericardium membrane. Clinical and cone beam computed tomography (CBCT) radiographic assessments of the two groups were carried out after a 6-month follow-up period. *Results*: Clinically, the results showed a significant reduction in furcation involvement (F). The CBCT assessment also revealed reductions in depth (D), height (H), width (W), and 3D radiographic volume of furcation involvement in all study groups at baseline and at 6 months postoperative (*p* < 0.05) with no significant differences between groups. *Conclusions*: According to the results of the current study, a two-layer membrane formed by direct loading of bone graft particles onto a pericardium membrane can be used as an effective, reliable, and easy-to-use substitute for direct bone graft application into periodontal defects.

## 1. Introduction

Periodontitis is an inflammatory and destructive disease that affects periodontal structures and causes periodontal attachment and bone loss, leading to involvement of the furcation and, frequently, loss of molars [1].

According to the 2017 World Workshop on the Classification of Periodontal and Peri-Implant Diseases and Conditions, periodontitis can be classified into four stages according to the severity of periodontal destruction: stages I and II represent mild and moderate periodontitis; stage III is characterized by severe periodontal destruction with bone loss to the middle third of the root length or beyond; and stage IV represents the most advanced stage of periodontitis, involving extensive tooth loss and potential for complete loss of dentition [1].

A classification of stage III periodontitis reflects a state of severe destruction of the periodontium that involves severe clinical attachment loss of 5 mm or more, deep periodontal pockets, severe bone loss reaching to or beyond the middle of the roots, and class II or III furcation involvement [1].

Careful clinical and radiographic assessments of furcation defects are critical for proper diagnosis and treatment. By means of clinical assessment using a Nabers probe and radiographic assessment using 2D methods, such as periapical or panoramic radiography, or the 3D method of cone beam computed tomography, a proper diagnosis of furcation assessment can be achieved [2].

Furcation defects can be classified using any one of several classification systems, depending on the severity of furcation involvements and whether their directions are horizontal or vertical. The Hamp classification for horizontal furcation involvement includes three classes. For class I, the depth of furcation involvement is less than 3 mm; for class II, the depth of furcation involvement is 3 mm or greater but is not through-and-through; for class III, there is complete bone loss within the furcation and the defect is through-and-through [3].

Radiographs are essential for examination, diagnosis and decision making in periodontal treatment as they provide information that cannot be obtained by clinical examination, such as the extent of remaining bony support and the morphology of bone defects. Cone beam computed tomography (CBCT) images exhibit a high level of accuracy in assessing furcation involvement. The use of CBCT radiography for furcation assessment offers the advantage of 3D analysis of the shape, size, morphology, and severity of furcation defects, by means of different views—including sagittal, axial, coronal, and 3D—which cannot be provided by conventional radiographic methods that have low sensitivity for furcation defect detection [4].

Different treatment modalities can be used for furcation defects, ranging from scaling and root planing to open flap debridement, resective osseous surgery, and guided tissue regeneration (GTR) [3]. In GTR, a barrier membrane is used to completely cover and surround the periodontal defect, to prevent the apical migration of the epithelium and gingival connective tissue of the flap to the defect, and also to maintain space under the membrane, giving time for bone and new periodontal ligaments to regenerate [5]. A bone graft used in association with GTR can then serve as a scaffold or a filler to stabilize the blood clot, maintain space for regeneration, and prevent the collapse of the membrane [6].

Bone grafts can be classified into autografts, allografts, xenografts, and alloplastic materials. Among these, autografts are considered the gold standard on account of their high osteogenic characteristics relevant to bone healing and regeneration. However, they also involve the disadvantages of pain, bleeding, and donor site morbidity. Allografts and xenografts are alternative options, but these have major limitations associated with rejection, disease transmission, and cost [7].

An alloplastic bone graft is a synthetic and inert bone graft substitute with exclusively osteoconductive properties. Such materials eliminate the risk of disease transfer and other complications associated with allografts and xenografts, and can be further categorized as bioactive glass, polymer, nonceramic, beta-tricalcium phosphate, or hydroxyapatite. Alloplasts act primarily to maintain space and exhibit exclusively osteoconductive properties [8].

The nanoscale features of nanohydroxyapatite provide synthetic bone grafts with more advantages, compared with conventional bulk material, due to their higher surface area, quicker resorption time, closer contact with surrounding tissues, easier preparation and synthesis, and more controlled physicochemical properties with the same degree of biocompatibility as conventional material [9].

GTR for class II furcation defects can be carried out using either a combined bone graft or a barrier membrane alone. A bone graft results in a more beneficial regeneration, with greater blood clot stabilization and decreased dead space beneath the barrier membrane. Despite this, many controlled clinical studies have shown that combining bone grafts with barrier membranes do not provide any additional benefit, compared with the GTR procedure alone; indeed, it may result in additional complications, such as graft containment, epithelial exclusion, microbial contamination, and variable inductivity of the graft [10].

Resorbable and nonresorbable barrier membranes have recently been introduced in GTR, and the use of resorbable membranes for GTR in human intrabony defects is now increasing [10]. Collagen membranes are widely used because they offer a number of advantageous properties. Collagen promotes vascularization, or the formation of new blood vessels. In addition, it activates osteoblasts, thereby promoting osteogenesis. It also facilitates wound healing, and exhibits antimicrobial properties [10].

Bovine pericardium membrane promotes tissue regeneration as it induces the periodontal ligament fibroblasts, and proliferation and regeneration of osteoblasts, by providing a naturally compatible microenvironment for host cell migration and proliferation. This is because of the various advantages of bovine pericardium membrane, which include acellularity, good consistency, and excellent mechanical properties—with a minimum thickness of 0.5 mm enabling dependable suture retention and ideal operative handling characteristics [11].

A composite, sandwich-structured, multilayered membrane consisting of a top layer of collagen and a bottom layer of hydroxyapatite provides a low-cost barrier membrane for GTR with good flexibility, good mechanical properties, and high bioactivity [12]. The addition of nanocarbonated hydroxyapatite to the porous side of a three-layered membrane of nanocarbonated hydroxyapatite/collagen/poly(lactic-co-glycolic acid) improves both the biocompatibility and the osteoconductivity of the membrane and allows for 3D cell ingrowth and differentiation [13].

Because of the previously mentioned drawbacks of bone graft materials, and because the use of bone grafts to support barrier membranes has been found to be of limited clinical significance in controlled studies, we sought in this study to clinically and radiographically evaluate the efficacy of a two-layered membrane formed by direct loading of nanohydroxyapatite particles over a pericardium membrane, and compared this with direct application of a nanohydroxyapatite bone graft, in treatment of stage III periodontitis.

## 2. Materials and Methods

Forty patients aged between 39 and 65 years who were suffering from stage III periodontitis with class II buccal furcation defects were selected from the Periodontology Clinic, Faculty of Dentistry, Tanta University, Egypt. The sample size was calculated using power analysis by Epi-Info software package created by the World Health Organization (WHO) and Center for Disease Prevention and Control (CDC) version 2007. The confidence limit was 95%, power of the study was 80%, case to control ratio was 1:1, and the sample size was found to be *n* = 40 (20 in group 1 and 20 in group 2) with an extra 9 defects to avoid sample attrition. In order to include the patients in this study, their consent was obtained, and all procedures were explained before the start of treatment. The study was ethically approved by the research ethics committee on 3 December 2020, with reference No. TUCDEEC/03122020/MOElboraey.

To be included in the study, patients were required to be suffering from class II furcation defects (F) with ≥3 mm horizontal probing depth and the gingival margin coronal to the level of the roof of the furcation [3], probing depth (PD), and a clinical attachment level (CAL) ≥ 5 mm. Patients with mandibular first or second molars, those with root caries or endodontic treatment, as well as smokers, diabetics, pregnant or lactating women, and those who had used antibiotic agents in the previous 6 months were all excluded from the study.

The furcation defects were divided into two treatment groups of equal size:

- Group I was treated with a two-layered membrane consisting of a pericardium membrane with nanohydroxyapatite particles prepared using the casting evaporation technique [14].
- Group II was treated with direct application of a nanohydroxyapatite bone graft into the furcation defect covered with pericardium membrane prepared using the casting evaporation technique [14] (NanoTech Egypt for Photo-Electronics (NTE), Cario, Egypt).

### 2.1. Study Design

#### 2.1.1. Clinical Assessment

PD and CAL were measured by a Williams probe. Customized acrylic stents were used for standard and fixed angulation and for insertion of the periodontal probe at baseline and at 6 months following therapy [15]. Furcation assessment was measured using a graduated Naber’s probe at each surgical site before surgery (baseline), during surgery, and at 6 months after surgery [3].

#### 2.1.2. Radiographical Assessment

**Figure 1:**
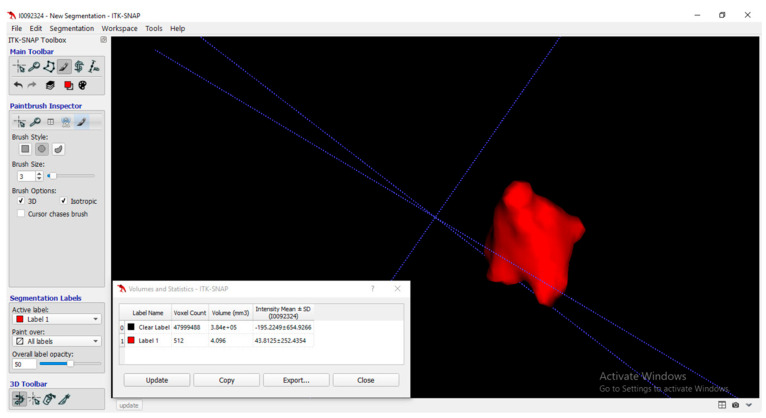
Three-dimensional volume analysis using ITK-SNAP software.

Periapical radiographs were taken during examination for detection of radiographic furcation involvement. Using cone beam computed tomography (CBCT), 3D volume of buccal furcation was measured at baseline, and at 6 months after treatment, using ITK—snap (ITK-SNAP Pro software, Materialise Dental, Bethesda, MD, USA) software version 3.8.0 (Figure 1). The height (H) and width (W) of furcation and root trunk (T) were measured using sagittal sections of CBCT, while the depth of furcation (D) was measured using axial and coronal sections of CBCT [2].

All pre- and postoperative CBCT scans were taken by a single trained technician with fixed radiographic parameters of exposure time, kilovoltage, milliamperes, field of view and voxel size.

### 2.2. Procedures

Patients or sites in the same patient were randomly selected using sealed envelopes and were then assigned to the appropriate group.

**Figure 2:**
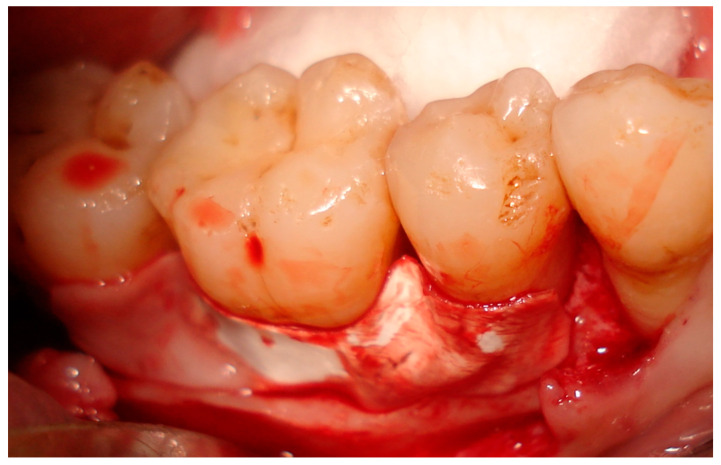
Third-generation pericardium membrane adaptation over a furcation defect.

Prior to surgery, all patients received repeated scaling and root planing as well as comprehensive oral hygiene instructions, with re-evaluation after one month. A surgical procedure (regenerative surgical flap) was then carried out. The treatment protocol was similar to that described by Mengal et al. (2006) [16], as follows:

- Presurgical combination antibiotic therapy was provided, consisting of Amoxicillin/clavulanate 375 mg tablets and Metronidazole 250 mg tablets three times per day, with anti-inflammatory systemic medications prescribed to patients the day before surgery.
- The surgical site was anaesthetized using the infiltration technique. Full-thickness intrasulcular incisions were carried out around the tooth to be treated, extended by one tooth mesial and one tooth distal, so far as possible according to condition.
- Complete debridement and degranulation of the defect was carried out, with additional scaling and root planning of defects.
- The granulation tissue on the inner side of the flap was curetted. Surgical areas were carefully rinsed with sterile saline, and isolated with cotton rolls.
- In group I patients, the pericardium membrane with nanohydroxyapatite particles on its surface was used alone without bone graft filler. The membrane was trimmed and adapted to cover the furcation defect and was extended from the cementoenamel junction (CEJ) to 3 mm beyond the defected furcation (Figure 2).
- In group II patients, the defects were filled with nanohydroxyapatite bone graft material, and the pericardium membrane was then adapted over the graft, extending from the CEJ to 3 mm beyond the defect.
- Flaps were sutured coronally to the original levels to cover the treated sites with soft tissue using 4-0 vicryl sutures. Simple interrupted sutures were used for flap closure.
- A simple modification at suture technique was made, as after making the knot for the interrupted suture, the surgical suture material was suspended over the contact areas then tied again with the end of the first knot.
- The surgical areas were then packed with periodontal dressing for two weeks.

Postoperative instructions were given for the use of 0.1% chlorhexidine mouthwash twice daily for two weeks, for completing the course of the antibiotic medication combination in one week, and for using anti-inflammatory and analgesic medications if necessary. These instructions were given to all participants. Periodontal dressing and suture removal were performed after 14 days. Supportive periodontal therapy was then performed monthly until the end of the study period.

## 3. Statistical Analysis

All data were organized and tabulated, and all necessary statistical analyses carried out, using the computer software Statistical Package for Social Science (SPSS version 23, Armonk, New York, NY, USA, IBM Corp.). Comparisons between the data of the two study groups during the different stages of the study were carried out using ANOVA testing.

### 3.1. Clinical Results

The results showed significant reductions in PD, CAL, and F in all study groups across the entire study period. Intergroup comparisons revealed no significant differences. However, while not statistically significant, group I patients exhibited more favorable results as compared with those in group II.

**Figure 3:**
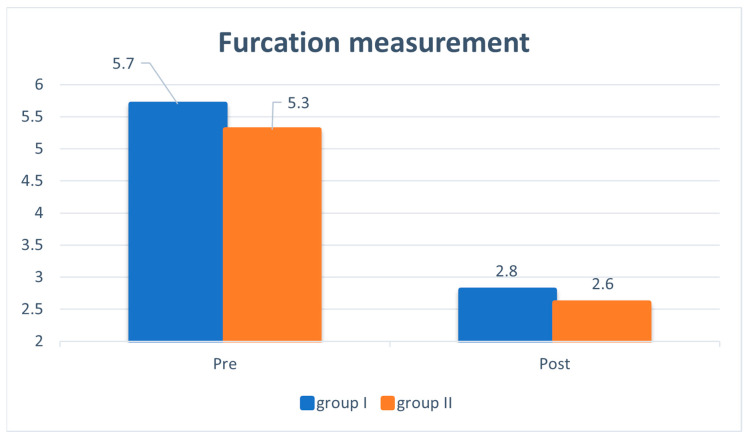
The mean furcation assessment values for groups 1 and 2 at baseline and at 6 months postoperative.

The significant reductions in PD, CAL, and F were revealed by the following measurements: for group I, the mean values of PD, CAL, and F decreased from 5.90 ± 0.38, 6.3 ± 0.62, and 5.70 ± 0.67 mm, to 2.70 ± 0.47, 2.90 ± 0.48, and 2.80 ± 0.58 mm, respectively; for group II, mean values of PD, CAL, and F decreased from 5.6 ± 0.47, 6.1 ± 0.49, and 5.3 ± 0.97 mm, to 2.7 ± 0.22, 3.1 ± 0.77, and 2.6 ± 0.23 mm, respectively (Table 1, Figure 3).

### 3.2. Radiographic Results

**Figure 4:**
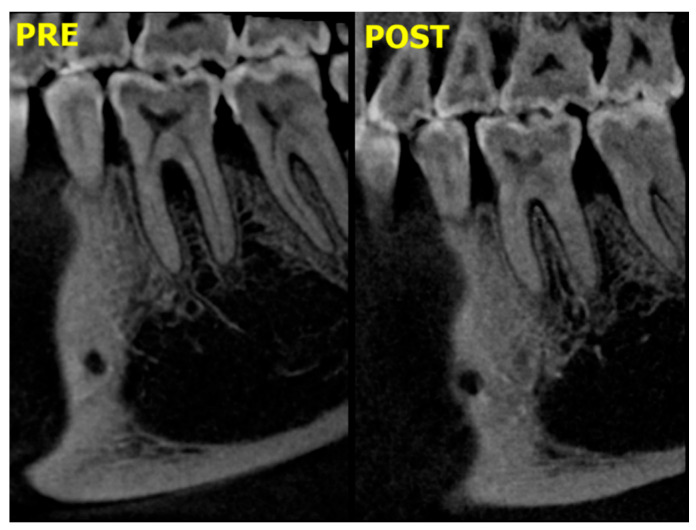
Sagittal view shows preoperative and postoperative furcation bone improvement.

The results shown in Figure 4 show a significant decrease in D, H, W, and 3D, as observed by CBCT, for all study groups at baseline and at 6 months postoperative, with no significant differences in intergroup comparisons for D, H, and W. However, the 3D volume assessment did reveal a significant difference between the two study groups, in favor of group I.

**Figure 5:**
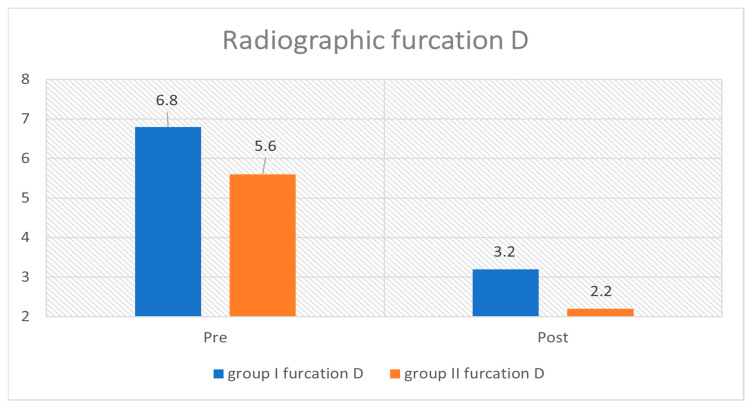
The mean radiographic furcation depth in mm values for groups 1 and 2 at baseline and at 6 months following surgery.

The mean values of furcation depth (D) and 3D furcation volume showed significant reductions across the study period (*p* < 0.05) for both groups. For group I, the mean values of D and 3D furcation volume measurements decreased from 5.33 ± 0.90 mm and 13.79 ± 4.45 mm^3^, to 1.70 ± 0.54 mm and 7.18 ± 2.8 mm^3^, respectively; for group II, the mean values of D and 3D furcation volume measurements decreased from 5.52 ± 1.10 mm and 6.23 ± 1.5 mm^3^, to 2.03 ± 0.69 mm and 3.99 ± 0.52 mm^3^, respectively (Table 2, Figure 5).

No significant differences were found when comparing the D, H, W, and T values for tooth furcation obtained during surgical procedures with radiographic measurement of the same parameters taken from baseline CBCT (*p* > 0.05). The mean values of D, H, W, and T at baseline using CBCT were 5.03 ± 1.67, 3.15 ± 0.66, 2.79 ± 0.86, and 2.89 ± 0.65 mm, respectively; during surgical assessment, values for the same parameters were 3.15 ± 0.66, 3.00 ± 0.67, 2.88 ± 0.60, and 2.50 ± 0.32 mm, respectively. The mean differences between surgical and baseline CBCT measurements were 0.28 mm for D, 0.15 mm for H, 0.09 mm for W, and 0.39 mm for T.

## 4. Discussion

The GTR concept is based on the use of a barrier membrane for preventing apical migration of epithelial cells, allowing more time for PDL and bone to regenerate [6]. Many factors can influence the predictability and the result of GTR of furcation defects, including anatomical factors, such as cervical enamel projection, enamel pearls, root concavities, root trunk length or bifurcation ridges, and accessory canals [17]. In this study, all defects that involved factors likely to affect a positive GTR outcome were excluded. All subjects were free of medical complaints, to avoid the possible impact of systemic disorders on the periodontal condition and also any possible effect on tested clinical parameters. This was because many systemic disorders have been implicated as risk factors or indicators for adverse periodontal conditions.

The present study was designed to evaluate the combination of bone graft and nanoscale materials over the pericardium membrane, forming a two-layered membrane for the treatment of grade II furcation involvement in patients with stage III periodontitis, based on the principles of GTR to avoid the drawbacks of direct application of bone into the furcation defect, and exploring the easy and rapid procedures of GTR.

Patients, or sites in individual patients, were randomly selected using sealed envelopes to prevent bias. The results of the present study showed significant reductions in PD, CAL, and F measurements in both study groups across the study period, but no significant differences between the groups.

The addition of nanohydroxyapatite produced more favorable results, as evidenced by the significant decreases in F, CAL, PD, and 3D volumes (Table 1 and Table 2, Figure 5). The better performance of nanoscale structures, compared with conventional bulk restoration, was the result of the inherited advantages of nanohydroxyapatite, including high surface area and quick resorption time (within 12 weeks) when used for the treatment of human intrabony defects [18].

A case series study conducted by Mathew et al. [19] on the treatment of grade II furcation defects using porous hydroxyapatite bone grafts revealed that this method led to significant reductions in PD, increases in clinical attachment levels, and reductions in furcation defect depth of 3.25 ± 1.28 mm, as well as increased bone gain at 6-month follow-up. These findings are in agreement with the results obtained for group II in our study, because we recorded a significant reduction in F measurements, with furcation assessment decreasing from 5.3 ± 0.97 mm to 2.60 ± 0.23 mm.

The pericardium membrane used in the current study was chosen because of the many favorable properties of collagen; its hemostatic property aids the formation of clots which adhere to the root surface and facilitate wound maturation, thereby enhancing regeneration. Once it is infiltrated by vascular channels, it forms a matrix for periodontal ligament fibroblast chemotaxis. Additionally, pericardium membrane can be easily shaped and adapted to the root surface due to its good biomechanical properties. Finally, it is a weak immunogen that is biodegradable, and thus requires no further surgery for removal [11].

The authors of [20] conducted a clinical and histological case series study to evaluate the application of pericardium barrier membrane in the treatment of intrabony defects. Their results revealed significant clinical improvements regarding PD, CAL, and bone gain at 1 and 3 years after surgery (*p* < 0.01). These findings are consistent with the results for group II in the current study. These patients were treated for intrabony furcation defect by means of a pericardium membrane with nanohydroxyapatite bone graft. Within this group, significant decreases in PD and CAL, from 5.6 ± 0.47 mm and 6.1 ± 0.49 mm, to 2.7 ± 0.22 and 3.1 ± 0.77 mm, respectively, were recorded.

One meta-analysis of 21 randomized clinical trials on patients with chronic periodontitis sought to compare the effects of GTR with and without bone grafts, in the treatment of intrabony defects. The results showed a nonsignificant gain in CAL of 0.32 mm, and an intergroup comparison which favored GTR treatment with bone graft. These findings are also comparable with the results of the current study, in which CAL decreased from 6.3 ± 0.62 mm to 2.90 ± 0.48 mm in group I, where the two-layered membrane was used, and decreased from 6.1 ± 0.62 mm to 3.1 ± 0.77 mm in group II, where the nanohydroxyapatite was directly applied into the furcation defects, so that there was a nonsignificant difference between the two study groups [21].

Contrarily, the results of another meta-analysis that evaluated twenty randomized controlled clinical trials with at least 6 months of follow-up, and which compared GTR with and without bone graft for treatment of furcation II defects according to the Hamp classification [22], revealed a statistically significant reduction in furcation involvement depth, and in probing depth, with significant bone fill (vertical/horizontal). The GTR with bone graft produced results that were statistically significantly better, compared with GTR alone.

In the current study, the use of a collagen membrane with nanohydroxyapatite as a bone grafting material led to a significant decrease in PD, CAL, and F measurements in group II, with mean values for PD, CAL, and F decreasing from 5.6 ± 0.47, 6.1 ± 0.49, and 5.3 ± 0.97 mm, to 2.7 ± 0.22, 3.1 ± 0.77, and 2.6 ± 0.23 mm, respectively. These results are in agreement with the findings of Santana et al. [23], who studied the efficacy of collagen and hydroxyapatite in human mandibular class II furcation and found significant reductions in PD, CAL, and F, of 3.65 ± 0.6 mm, 3.05 ± 0.6 mm, and 3.45 ± 1.3 mm, respectively.

Multiple-layer membranes are considered one of the third-generation barrier membranes now used in GTR. The two-layered membrane used for group I of this study consisted of a pericardium layer and a nanohydroxyapatite layer, a composite which enables cell adhesion, attachment, and proliferation in a three-dimensional fashion, on account of its high surface area and 3D porosity system [14].

Talam et al. [24] added chitosan and collagen to nanohydroxyapatite particles to form collagen chitosan membrane (a two-layered membrane). They then used this for treatment of class II furcation defects and compared its performance with that of nanohydroxyapatite bone graft alone, without barrier membrane. When compared to baseline, an intragroup comparison revealed statistically significant reductions in PD, CAL, and F, and significant radiographic bone gains, in both test and control sites. In the current study, 3D barrier membranes were used either alone (group I) or with nanohydroxyapatite bone graft with pericardium membrane (group II) for the treatment of class II mandibular furcation defects. The results of the current study showed that the combination of pericardium and nanohydroxyapatite as one membrane (group I) produced statistically significant improvements in different clinical parameters such as PD, CAL, and F, as well as significant bone gain. In addition, when the study groups were compared, group I exhibited statistically significant radiographic bone gain, in terms of 3D volume.

The statically significant results for group I could be explained by the addition of nanohydroxyapatite, which seemed to improve the biocompatibility, osteoconductivity, and biomechanical properties of the multilayered membrane. By such means, direct implantation of bone graft into the bone defect was avoided, thereby preventing the inflammatory response that results when hydroxyapatite is implanted within connective and bone tissues. Histological studies have demonstrated that healing often occurs with encapsulation of hydroxyapatite graft materials in connective tissue, with no indication of new periodontal attachment, osteogenesis, or cementogenesis in the host tissues adjacent to the graft particles.

Radiographic evaluation offers a noninvasive method for assessing changes in hard tissues. For this reason, it was employed in our study, with CBCT radiographs taken at baseline and at 6 months after treatment.

Today, CBCT is one of the most important radiological diagnostic modalities in dentistry, because it is widely available, easy to use, and offers high accuracy, especially in the diagnosis and determination of treatment plans for furcation involvements, for which CBCT can provide comprehensive assessments of furcation defects in coronal, sagittal, axial sections, and also in three dimensions. With conventional two-dimensional radiography [8], it is difficult and sometimes impossible to detect any changes during radiographic interpretation of the furcation defect when using panoramic or periapical techniques, during GTR follow-up periods [25].

In the present study, CBCT results showed significant reductions in D, H, W, and 3D in both study groups at baseline and at 6 months postoperative, with no significant differences when the groups were compared. However, a 3D volume assessment using CBCT revealed a more favorable outcome for group I than group II.

The results of the current study show that CBCT assessment for furcation can be an effective substitute for surgical re-entry, as we found no significant difference between the CBCT assessment and the intrasurgical assessment. This finding is in agreement with the results obtained by Suh et al. [25] who measured depths of class II furcation defects using both surgical assessment and CBCT assessment, and concluded that CBCT assessment can be used as a proper substitute for open flap assessment at bone level.

The present study found no significant differences between CBCT and intrasurgical furcation assessment, in terms of D, H, W, and T values for both study groups. For group I, the mean vertical height and the depth of the furcation measured intrasurgically were 4.13 mm and 4.07 mm, respectively. Using CBCT, the corresponding values for the same group were 4.05 mm and 4.06 mm. For group II, the height and depth measured intrasurgically were 4.53 mm and 4.6 mm, respectively; using CBCT, the corresponding figures were 4.6 mm and 4.45 mm. These results agree with those obtained by Gupta et al. [2], who used CBCT for radiographic evaluation of GTR in the treatment of mandibular Class II furcations.

Based on the results of the current study reported above, it can be claimed that clinical and radiographic outcomes improved for all tested groups. The combination of pericardium with nanohydroxyapatite, whether applied into the furcation itself as a bone graft or incorporated with the membrane, may have had a potentiating effect on both clinical and radiographic outcomes. However, given a choice between GTR with or without graft material, we would choose the multilayered membrane, because it was found to be easily and rapidly applied, as well as low in cost.

In conclusion, further studies on the usage of two-layered membrane of pericardium membrane and nanohydroxyapatite graft material in the treatment of advanced classes of furcation defects and other intrabony defects may be of value, especially with more modification of the biomechanical properties of the membrane to avoid the membrane collapse during surgical insertion, which has been encountered in some cases. In the light of our study’s findings, we conclude that pericardium membrane may be an important potentiating element, when combined with nanohydroxyapatite graft materials, resulting in more favorable clinical and radiographic outcomes.

## Figures and Tables

**Table 1:** The mean furcation assessment (F in mm) values in groups 1 and 2 at baseline and 6 months postoperative.

F	Group 1	Group 2	Repeated Measures ANOVA *p* Value
At baseline:			
Mean ± SD	5.70 ± 0.67	5.30 ± 0.97	0.058 ns
At 6 months:			
Mean ± SD *p*	2.80 ± 0.58 0.000	2.60 ± 0.23 0.000	0.053 ns

**Table 2:** The mean 3D furcation volume assessment (F in mm^3^) values for groups 1 and 2 at baseline and at 6 months following surgery.

Groups	Volume		t *p*-Value
	Pre	Post	
Group I	13.79 ± 4.45	7.18 ± 2.8	0.002
Group II	6.23 ± 1.5	3.99 ± 0.52	0.008
*p*-value	0.003 *		

* Significant Difference.

## Data Availability

Data is unavailable due to privacy restrictions.
